# Effect of Structural Design of Silver/Silver Chloride Electrodes on Stability and Response Time and the Implications for Improved Accuracy in pH Measurement

**DOI:** 10.3390/s90100118

**Published:** 2009-01-07

**Authors:** Paul J. Brewer, Richard J. C. Brown

**Affiliations:** Analytical Science Team, National Physical Laboratory, Teddington, Middlesex, TW11 0LW, UK; E-mail: richard.brown@npl.co.uk

**Keywords:** pH, Harned cell, Ag/AgCl chloride electrode, stability, primary methods

## Abstract

The response time of thermal electrolytic Ag/AgCl reference electrodes is defined by the porous structure that limits the rate at which traces of any previous solution are diluted by any new solution environment. The electrode stabilisation time when transferred from one electrolyte to another has been shown to change when different structural designs to the conventional sphere of Ag/AgCl are used. Electrodes fabricated with cylindrical architectures of Ag/AgCl have shown improved stability and reach equilibrium faster than spherical electrodes. This work has positive implications for the accuracy and throughput of primary pH measurements made using the Harned cell.

## 1. Introduction

The concept of pH was first introduced by SØrensen in 1909 [1]. Over the past century, measurements of pH have become arguably the most numerous analytical measurements made [2-5]. Examples of areas where pH measurements are frequently made involve chemical and drug manufacture, blood gas determinations, effluent discharge control, food and drink processing and water purity. It is therefore essential to ensure the validity and traceability of these measurements [6, 7].

Primary pH standard values are determined from an electrochemical cell arrangement known as the “Harned cell” which does not contain a liquid junction and relies on well characterised Ag/AgCl reference electrodes for operation [8]. The Harned cell has the potential to be a primary method for the absolute measurement of pH, providing that it can conform to the accepted definition of a primary method [9] that requires a methodology and operation that can be completely described and understood, for which a complete uncertainty statement can be written down in terms of SI units.

The long and short term stability of Ag/AgCl reference electrodes is of paramount importance for accurate pH measurement as a small change in the potential of a Ag/AgCl reference electrode makes a significant contribution to the measurement uncertainty. Therefore in an attempt to select a set of similar reference electrodes and reduce the risk of changes in potential between electrodes, it is common practice to reject individual reference electrodes which differ from the average of the group by more than 100 micro volts [4]. The stability of the potential when Ag/AgCl electrodes are transferred between different electrolytes was first commented upon by R. G. Bates in the 1940s and has since been re-investigated by K. W. Pratt [10]. Thermal electrolytic type Ag/AgCl reference electrodes comprising a sphere of Ag/AgCl on a Pt wire are the conventional choice for use in the Harned cell. These electrodes are often stored in solution of 0.01 M HCl when not in use and are transferred to Harned cells containing 0.1 M HCl or buffer solutions (with added chloride) when required for measurement. The transfer of an electrode between solutions induces a large initial change in the reference potential (as compared to a Ag/AgCl reference electrode which has been allowed enough time to reach equilibrium in a new solution). This initial change then decays as the Ag/AgCl reference electrode reaches an equilibrium potential in the new solution environment. These shifts in electrode potential can have significant implications for the accurate operation of the Harned cell and the throughput of the certification of primary reference buffers. Previous work by Brown *et al.* [11] investigated the timescale of the equilibrium process. They reported the effect of the diameter of the Ag/AgCl sphere used in the electrodes on the equilibration time and suggested the presence of a microporous structure that limits the rate at which traces of any previous solutions are diluted by any new environment. Larger diameter spheres of Ag/AgCl were shown to require longer times to reach equilibrium which is consistent with the process being described by diffusion whereby traces of the previous solution diffuses out of the pore structure while the new solution diffuses in.

At present, little data exists for the long term stability of Ag/AgCl electrodes. This paper investigates the role of the Ag/AgCl structure on the short and long term electrode stability. Characterisation of electrodes prepared by the standard thermal electrolytic procedure has been compared to two alternative manufacturing processes. The effect of changing the structural design away from the conventional sphere of Ag/AgCl in thermal electrolytic type electrodes has also been investigated with novel cylindrical and planar architectures. This work has resulted in a proposed new structure for the Ag/AgCl electrode with improved stability and response time and is likely to have positive implications for the accurate operation of the Harned cell.

## 2. Results and Discussion

**Figure 1.**
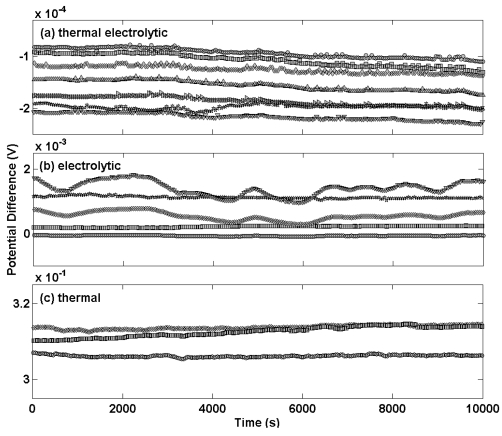
Transient potential difference measurements for Ag/AgCl electrodes equilibrated in a 0.01 M HCl solution. Each profile corresponds to an individual electrode. Electrodes were prepared using (a) a thermal electrolytic procedure, (b) an electrolytic procedure and (c) a thermal procedure.

Figure 1 shows the differential potential transients for a selection of Ag/AgCl reference electrodes prepared by the three different procedures compared to a thermal electrolytic Ag/AgCl electrode used as a defacto reference (a different scale for the y-axis scale is used for each sub plot). Electrodes manufactured by the most extensively used thermal electrolytic method are presented in Figure 1(a).

The transients of these electrodes display good long term stability and deviate in each case by no more than 40 μV across the measurement period of approximately 3 hours (chosen to represent the time period typically employed for making Harned cell measurements of pH). The transients are highly repeatable and meet the specification for use in the Harned cell with the largest disagreement between any pair of electrodes being approximately 100 μV. The potential transients for electrodes prepared by the electrolytic method in Figure 1(b) exhibit comparatively poor long term stability with variations in potential of up to 800 μV for the most unstable electrode across the same measurement period. The repeatability is equally poor with the largest disagreement between any pair of electrodes being approximately 2 mV. The magnitude of the potential difference of the electrolytic type electrodes differs from the thermal electrolytic type by an order of magnitude. This high bias in potential of electrolytic type electrodes has previously been reported and attributed to strains and probable impurities introduced in the drawing process of the Ag wire used to produce the electrodes, which might also account for the variability in potential between electrodes [12].

An explanation for the comparatively poor long term stability of the electrolytic type electrodes can be offered by considering the low geometric surface area and the exchange current density (that is a fundamental characteristic of electrode behaviour and can be defined as the equilibrium rate of oxidation or reduction at an electrode expressed in terms of current) resulting in a relatively small absolute exchange current available to robustly define the equilibrium voltage. The structure of anodically formed silver halide films was first investigated by Huber with electron microscopy [13] and has since been reviewed and reported by Janz *et al* [14]. In Huber's work high purity silver sheets were anodized at current densities less than 10 mA/cm^2^ using 0.1 M halide solutions (which is analogous to the preparation of the electrolytic electrodes in the work here). Electron microscopy showed a structure with little porosity and with no pores penetrating entirely through the silver halide to the metal. These halide layers were classed as ‘locked’ surfaces. Janz also proposed alternative ‘porous’ and ‘open’ structures containing a higher degree of porosity and assigned these structures to AgCl layers prepared by thermal electrolytic means as a result of starting with a porous Ag_2_O paste. Hence thermal electrolytic type electrodes will result in a AgCl film with a higher geometric surface area. This implies a lower absolute exchange current at equilibrium for the electrolytic type electrode and hence compromises the long term stability. Impurities on the metal surface also affect the exchange current of the electrode. Since the thermal electrolytic electrodes were prepared from a Ag_2_O paste of higher purity than the silver wire used for the electrolytic electrodes this may also explain the poorer long term stability observed for the latter.

Electrodes manufactured with the thermal method [Figure 1(c)] exhibit a large potential difference with respect to the *defacto* reference and poor repeatability. Reference electrodes manufactured using this procedure are therefore unsuitable for Harned cell measurements. For this reason no further characterisation of these electrodes was carried out.

**Figure 2.**
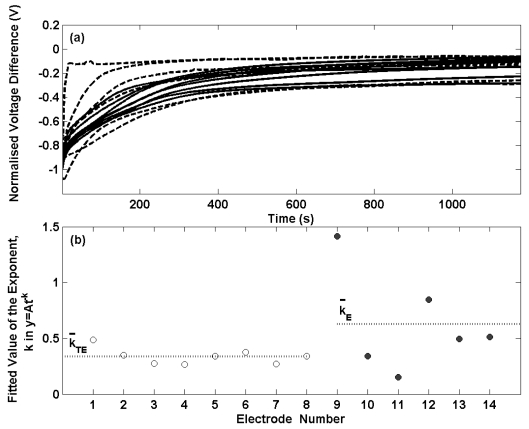
(a) Selected transient potential difference measurements for thermal electrolytic (solid lines) and electrolytic (dashed lines) after transfer from a 0.01 M HCl solution to a 0.025 M Na_2_HPO_4_/ 0.025 M KH_2_PO_4_ buffer solution. The electrodes were equilibrated in the 0.01 M HCl solution prior to transference. (b) The value of the exponent, k, in the power function of type y = At^-k^ fitted to the first 100 s of the transient potential difference measurements. The thermal electrolytic and electrolytic electrodes are represented by open and filled circles respectively. The dotted line in each case indicates the mean of the exponents determined for each electrode type.

Figure 2(a) presents the potential transients for the thermal electrolytic (solid lines) and electrolytic (dashed lines) electrodes shown in Figures 1(a) and (b) after transfer from a 0.01 M HCl solution to a 0.025 M Na_2_HPO_4_/0.025 M KH_2_PO_4_ buffer solution. The data has been normalised at t = 0 for each transient. For each electrode the potential differences are initially large and decay with time. The initial decay in potential of the electrolytic type electrodes is considerably more variable and less repeatable than the electrodes prepared by the thermal electrolytic procedure which in agreement with the long term stability measurements in Figure 1. Two of the electrodes prepared by the electrolytic process exhibit a swift decrease in potential after transfer to the buffer solution and achieve equilibrium after about 200 s. The remaining four electrolytic type electrodes display similar transients to the thermal electrolytic batch with longer times required to reach equilibrium. This can be seen more clearly in Figure 2(b) where the observed decay with time for the first 100 s (the time period in which a significant rate of change of potential is observed) has been fitted to a power function of the type (y = At^-k^) where t is the time elapsed in seconds, y is the normalised voltage difference and A is the normalised voltage difference when t = 0. Only data for the first 100 s has been considered here as the majority of the progress towards achieving equilibrium occurs in this time period hence making comparisons between electrodes clearer than with the data after 100 s. A power function was selected as this provided the best fit to the data. The exponents for the thermal electrolytic electrodes are represented with open circles and show good repeatability with a standard deviation of 0.07. The rate constants determined for the electrolytic type electrodes represented by filled circles show much larger variability between electrodes with a standard deviation of 0.45. The mean exponent for each group of electrode types is indicated by a dotted line and is greater for the electrolytic electrodes (mean exponent for electrolytic is 0.63 compared to a mean exponent for thermal electrolytic of 0.34). This demonstrates that electrodes fabricated by the electrolytic procedure on average achieve equilibrium at a faster rate than electrodes prepared by the thermal electrolytic method. This observation is consistent with the different structures of the two electrode types.

The porous structure of thermal electrolytic type limits the rate at which the electrodes can equilibrate, as any previous solution contained within the pores of the Ag/AgCl must diffuse out before a stable potential can be achieved. The structure of the electrolytic type however containing a less porous ‘locked’ structure significantly reduces the degree to which a previous solution is trapped and therefore results in faster equilibrium times when placed in a new solution.

**Figure 3.**
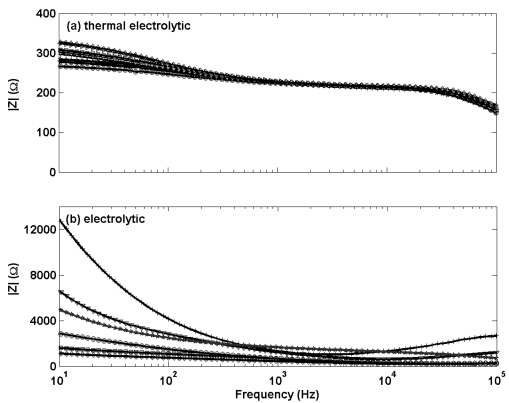
The impedance modulus |Z| as a function of the modulation frequency for electrochemical impedance measurements (modulation amplitude, 10 mV (RMS)) performed at open circuit potential in 0.01 M HCl for Ag/AgAgCl electrodes of (a) thermal electrolytic type and (b) electrolytic type.

Electrochemical impedance measurements in Figure 3 (a different scale for the y-axis scale is used for each sub plot) show the thermal electrolytic electrodes [Figure 3(a)] display lower and less variable resistive characteristics than the electrolytic type electrodes [Figure 3(b)]. There is no significant change in shape of the impedance response for either electrode type, suggesting a negligible difference in the structure of Ag/AgCl between electrodes fabricated by each method. However there is a noticeable difference in the shape of the impedance profiles when comparing between the two electrodes types. The thermal electrolytic type decay more steadily from an initially lower impedance response and continue to decrease throughout the frequency range of the measurement. The electrolytic type electrodes exhibit an initially large impedance response which decays until a plateau is reached at around 1000 Hz. The lower impedance response for the thermal electrolytic type electrodes can be explained by the porous structure present which facilitates conduction through the pores. The higher impedance response for the electrolytic type suggest the presence of a structure of lower porosity resulting from electrochemically converting the Ag wire to AgCl.

**Figure 4.**
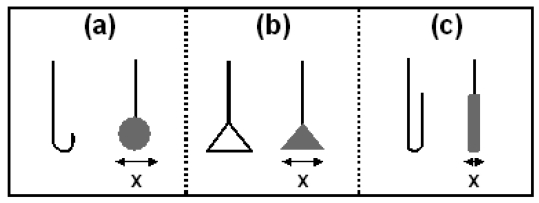
The wire skeleton (left of each pair) and added Ag/AgCl architecture (right of each pair) for the three structural designs of thermal electrolytic type electrodes; (a) the literature based spherical design, (b) the planar design, (c) the cylindrical design.

The ability of an electrode to adapt to a new environment on a short time scale is key to accurate Harned cell measurements as it increases the probability that Ag/AgCl electrodes will be at equilibrium within the typical timescales employed for Harned cell measurement. In addition a faster stabilisation time for electrodes is a result of a lower degree of porosity in the Ag/AgCl material and hence reduces the risk of contaminating the solution to be measured by transference of a previous solution. Although the electrodes prepared by the electrolytic method demonstrate on average a shorter time required for equilibrium in the new solution they display poor repeatability with a large deviation in rate constant between electrodes. Hence this alternative fabrication technique is inadequate when fabricating electrodes for Harned cell use and illustrates the virtue of using the thermal electrolytic procedure to establish a stable reference potential. Therefore in order to optimise the stability of Ag/AgCl electrodes, structural variations of the thermal electrolytic type structure are required to reduce the equilibration time after transfer to a new solution. Figure 4 shows a schematic of the three different Ag/AgCl architectures [(a) spherical, (b) planar and (c) cylindrical] with the skeleton of the Pt wire prior to adding the Ag_2_O paste on the left and the completed electrode on the right. The width of each electrode was determined using Vernier calipers around the axis marked ‘x’.

**Figure 5.**
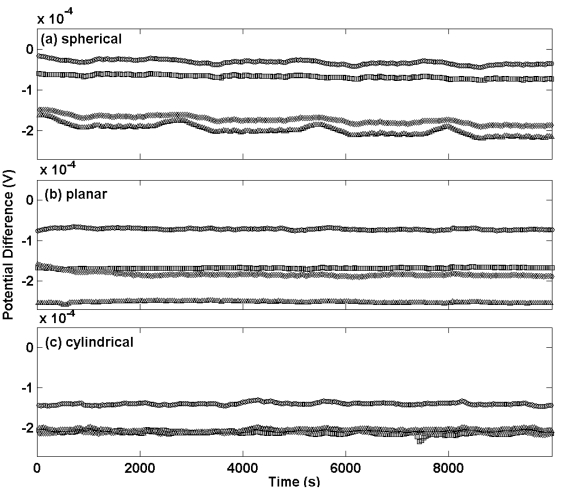
Transient potential difference measurements for thermal electrolytic type Ag/AgCl electrodes equilibrated in a 0.01 M HCl solution. Electrodes were prepared with three different Ag/AgCl structures of: (a) spherical design, (b) planar design and (c) cylindrical design.

Figure 5 shows the potential transients plotted on the same scale for thermal electrolytic type electrodes of each different Ag/AgCl architecture at equilibrium in a 0.01 M HCl solution. Potential transients for electrodes with a sphere of Ag/AgCl are presented in Figure 5(a) and exhibit almost identical behaviour to the batch shown in Figure 1(a). The largest discrepancy in potential difference between any two electrodes is about 150 μV and the drift over the time period for which this measurement has been carried out is negligible. Potential transients for planar and cylindrical Ag/AgCl architectures are shown in Figures 5(b) and 5(c), respectively. These transients almost replicate those of the spherical electrodes and indicate that the architecture of the Ag/AgCl mass has a minor if not negligible effect on the long term stability at equilibrium. This is expected since the Ag/AgCl architecture in each case was prepared using the same procedure and hence is likely to contain a porous structure of similar geometric surface area.

**Figure 6.**
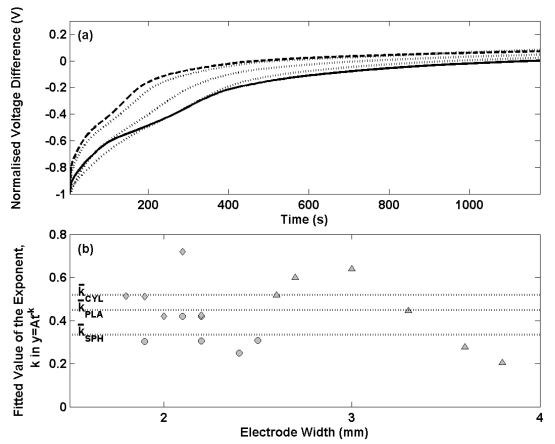
(a) Selected transient potential difference measurements for spherical (solid line), cylindrical (dashed line) and planar (dotted lines) AgAgCl architectures after transfer from a 0.01 M HCl solution to a 0.025 M Na_2_HPO_4_/ 0.025 M KH_2_PO_4_ buffer solution. The electrodes were equilibrated in the 0.01 M HCl solution prior to transference. (b) The value of the exponent, k, in the power function of type y = At^-k^ fitted to the first 100 s of the transient potential difference measurements. The spherical, cylindrical and planar designs are represented by the filled circles, diamonds and triangles respectively. The dotted line in each case indicates the mean of the exponents determined for each Ag/AgCl structure.

Figure 6(a) shows the normalised voltage difference for a selection of thermal electrolytic type electrodes containing the three different Ag/AgCl architectures after transfer from 0.01 M HCl solution to a 0.025 M Na_2_HPO_4_/0.025 M KH_2_PO_4_ buffer solution. The transients have been normalised at t = 0. A single representative transient for the spherical (solid line) and cylindrical (dashed line) electrodes is shown for clarity as each set of electrodes exhibited very similar potential transients. Three potential transients for the planar electrodes (dotted lines) are shown to present the broader variability observed as a result of a wider distribution of the width of Ag/AgCl on the Pt wire. The transient signature for each Ag/AgCl architecture is similar showing a dramatic initial decrease in potential difference that reduces with time. Electrodes with a cylindrical Ag/AgCl structure are shown to respond with a faster change in voltage difference compared to the conventional spherical type. A satisfactory equilibrium is achieved after 400 s for electrodes adopting the cylindrical Ag/AgCl structure whereas at least 800 s is required for the electrodes with a spherical Ag/AgCl architecture to reach the same status. Electrodes with a planar structure of Ag/AgCl exhibit variability across the set of six produced. One of the three examples in Figure 6 (a) is shown to perform similarly to the electrode with cylindrical Ag/AgCl architecture, another similarly to the spherical type and the third intermediate between the two.

Further electrode stability data is presented in Figure 6(b) for all electrodes prepared, where the decay in potential transient for the first 100 s has been fitted to a power function of the type (y = At^-k^) used in Figure 1(b). Exponents determined for the spherical, cylindrical and planar designs are shown with filled circles, diamonds and triangles respectively and are plotted against the width of the Ag/AgCl material on each electrode (dimension ‘x’ in Figure 4). The mean exponent is shown for each group of Ag/AgCl structures with dotted lines. The data shows electrodes with cylindrical Ag/AgCl architectures (mean exponent = 0.52) respond on a faster timescale than spherical (mean exponent = 0.35) and planar (mean exponent = 0.45) structures. The repeatability of exponents determined for each cylindrical type electrode compares well to spherical type electrodes of making them suitable for use in the Harned cell. The data also shows that both sets of electrodes have been prepared with similar widths of Ag/AgCl. For the planar type electrodes however, a broad distribution of electrode widths was employed (due to difficulties in reproducing the wire skeletons of the same dimension) which offers an explanation as to why the exponents are more variable. A correlation between electrode width and exponent occurs with wider electrodes responding more slowly to the new solution environment. This behaviour can be rationalised by considering the distance from the surface of the Ag/AgCl to the Pt wire. Electrodes with shorter pathways through the Ag/AgCl from the solution to the Pt wire are more likely to exhibit faster rate constants as a result of fewer pores available to trap the previous solution. The planar electrodes consist of a region of Ag/AgCl around the perimeter of the triangular skeleton with a short distance to the Pt wire and a region of Ag/AgCl inside the skeleton with a longer distance from solution to Pt wire. The time required for traces of any previous solution to diffuse from the porous structure is limited by the central region of this planar structure and hence results in a slower rate constant as the width of the Ag/AgCl in these electrodes is increased. This explains the increase in rate constant with the increased electrode width as the distance from the centre of the planar structure to the Pt wire becomes larger. The short equilibrium times for the cylindrical architectures result from a similar surface area to the spherical structure and a shorter distance from the surface to the Pt wire. This allows for faster equilibrium times as a result of fewer pores available to trap any previous solution. Although the planar electrodes have a larger surface area to assist diffusion of any trace of a previous solution in the porous structure, the mean exponents for the six electrodes is lower than for the cylindrical type due to the larger electrode widths.

**Figure 7.**
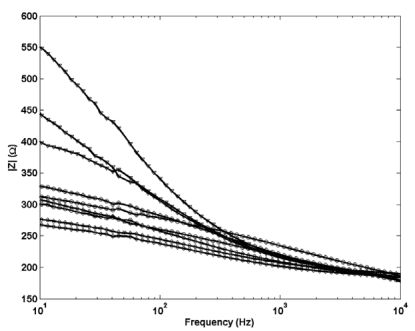
The impedance modulus |Z| as a function of the modulation frequency for electrochemical impedance measurements (modulation amplitude, 10 mV (RMS)) performed at open circuit potential in 0.01 M HCl for thermal electrolytic type Ag/AgAgCl electrodes. Selected data is presented with spherical, cylindrical and planar Ag/AgCl architectures represented by filled circles, filled diamonds and filled triangles respectively.

A selection of electrochemical impedance data for the three structural Ag/AgCl designs is presented in Figure 7. As expected no change is observed in shape between the profiles for each Ag/AgCl structure indicating the same porous structure in each. Electrodes with cylindrical Ag/AgCl structures are shown to be least resistive which supports their ability to equilibrate to a new solution on a faster timescale than the spherical and planar type which exhibit a higher impedance response. The resistance of the planar electrodes is significantly greater than the other two and is likely to result from the increased length of Ag/AgCl which the current needs to pass through to reach the Pt wire. This data confirms that the improved short term stabilisation in cylindrical electrodes can be directly related to the shape of the electrode and not simply the porous structure.

## 3. Experimental Section

The Ag/AgCl reference electrodes in this work were prepared by three different standard procedures outlined in the literature [14-19]. The first method involved the preparation of thermal electrolytic type Ag/AgCl reference electrodes which are the conventional choice and the most widely employed in Harned cell operation. This procedure was achieved by thermal decomposition (100 °C for 1 hour followed by 500 °C for 2 hours) of three separate applications of Ag_2_O paste to a Pt wire (99.99%, 0.5 mm diameter). Following this process, approximately 15% of the material was electrolytically converted to AgCl in a solution of 0.1 M HCl. The amount of Ag on each electrode was determined prior to anodisation and the charge required to convert 15% to AgCl was calculated. The appropriate current was then applied to achieve this charge over a 1 hour period. In addition to the conventional formation of a sphere of Ag/AgCl, thermal electrolytic electrodes were prepared with planar and cylindrical Ag/AgCl architectures of similar mass to investigate the effect of structural design on electrode performance (Figure 4). These electrodes were prepared with the same Ag_2_O paste and anodisation procedure employed for thermal electrolytic spherical architectures. In each case the Ag_2_O paste was applied to the different Pt skeletons to form the required architectures. A second method for preparing thermal type reference electrodes involved thermal decomposition (350 °C for 10 minutes) of three separate applications of a paste containing an amount fraction of 7 parts of Ag_2_O : 1 part of AgClO_3_ to the end of a Pt wire (99.99%, 0.5 mm diameter). Both of these methods result in an electrode consisting of a sphere of porous nature of approximately 85% Ag and approximately 15% AgCl. The third method to fabricate electrolytic type reference electrodes was achieved with a single anodisation step to convert approximately 15% of a Ag wire (0.5 mm diameter) to AgCl. A constant current density of 1 mA/cm^2^ of Ag was passed for 1 hour in a 0.1 M HCl solution. Prior to electrode preparation, the Ag and Pt wires were immersed in concentrated nitric acid to remove surface contaminants. Wires were then rinsed thoroughly with distilled water.

All solutions were prepared using 18.2 MΩ cm distilled and deionised water (MilliQ, Millipore) and ultra high purity chemicals (Fisher, UK). All experiments were carried out with solution temperatures of 20 ± 2 °C. All solutions were degassed with nitrogen before use (metrology grade, BOC UK). A positive pressure of nitrogen was maintained over the solution during experimentation.

Measurements to investigate the long term stability of the Ag/AgCl reference electrodes were carried out by using one of the thermal electrolytic Ag/AgCl electrodes as a defacto reference and placing it in a 0.01 M HCl solution with the other Ag/AgCl electrodes and allowing a period of at least twelve hours for equilibrium to be reached. All of the potentials are reported with respect to this electrode. In a separate experiment to test the timescale of the equilibration process, the defacto reference was placed in a solution of 0.025 M Na_2_HPO_4_/0.025 M KH_2_PO_4_ buffer and allowed to equilibrate for at least twelve hours. The electrodes under observation were then removed from the 0.01 M HCl solution and transferred to the phosphate buffer solution ensuring that any excess solution was removed. In both experiments, the potential difference between the electrodes and the reference was measured as a function of time using a high accuracy Keithley 2001 multimeter. Measurements were taken every 30 s and were acquired using software written in LabVIEW 7.1.

Impedance measurements were made using an Ecochemie Autolab PGSTAT 12, FRA module at open circuit potential with an applied ac perturbation of ± 10 mV (RMS) at 125 logarithmically spaced frequencies between 10 Hz and 100000 Hz. Experiments were performed in degassed 0.01 M HCl with a positive pressure of nitrogen maintained above the solution. A standard three electrode cell was used with a Pt flag counter electrode and an Ag/AgCl reference electrode connected to the main cell via a luggin capillary.

## 4. Conclusions

Characterisation of Ag/AgCl reference electrodes prepared by the standard thermal electrolytic procedure has been compared to two alternative manufacturing processes and has shown this electrode type to be the most repeatable and provide the most stable reference potential. However this work shows that on average thermal electrolytic electrodes are slower to respond to a new solution environment (mean exponent = 0.34) than electrodes manufactured by the electrolytic process (mean exponent = 0.63). This ability to adapt on a short time scale is crucial for making accurate Harned cell measurements as it increases the probability that Ag/AgCl electrodes will be at equilibrium within the typical timescales employed and reduces the risk of contaminating the solution to be measured by transference of a previous solution (as a result of trapping in the porous structure).

The response time of these highly repeatable and stable thermal electrolytic electrodes has been improved by changing the structural design away from the conventional sphere of Ag/AgCl. Electrodes with planar and cylindrical architectures were proposed. Electrodes with cylindrical Ag/AgCl structures displayed faster average response times (mean exponent = 0.52) compared to the spherical and planar type (mean exponent = 0.35 and 0.45 respectively). The fastest cylindrical electrode was shown to achieve equilibrium in half the time required for electrodes prepared with spheres of Ag/AgCl while still maintaining the same long term stability. This optimal structure is a result of a compromise between having enough redox material for long term stability of the reference voltage and not having too much, so that the thickness of Ag/AgCl inhibits the diffusion of a previous solution and reduces the response time. This proposed new cylindrical structure for the Ag/AgCl electrode could have positive implications for the accurate operation and increased throughput of the Harned cell. This is the first time such a design has been postulated and tested experimentally.
